# Tofacitinib extends survival in a mouse model of ALS through NK cell-independent mechanisms

**DOI:** 10.3389/fimmu.2025.1662197

**Published:** 2025-11-05

**Authors:** Lillia A. Baird, Samuel J. Teener, Ian F. Webber-Davis, Andrew D. Carter, Fang Huang, Dae-Gyu Jang, Joshua P. Famie, Caroline E. Piecuch, Kai Guo, Eva L. Feldman, Benjamin J. Murdock

**Affiliations:** ^1^ Department of Neurology, University of Michigan, Ann Arbor, MI, United States; ^2^ Graduate Program in Immunology, University of Michigan, Ann Arbor, MI, United States; ^3^ School of Medicine & Health Sciences, University of North Dakota, Grant Forks, ND, United States

**Keywords:** ALS, immune system, motor neuron disease, natural killer cells, neurodegeneration

## Abstract

**Background:**

Amyotrophic lateral sclerosis (ALS) is a lethal neurodegenerative disease with few treatment options, rendering the development of new, effective therapeutics of critical importance. The immune system plays a substantial role in ALS pathology, with multiple cell populations implicated in disease progression. Natural killer (NK) cells are innate immune cells that accumulate in the brain and spinal cord during ALS, increasing neuroinflammation and killing motor neurons. Depleting NK cells extends survival in mouse models of ALS. Tofacitinib, an FDA-approved janus kinase (Jak) and signal transducer and activator (STAT) pathway inhibitor, reduces NK cytotoxicity and decreases overall levels in peripheral blood and may represent a potential ALS therapy. Therefore, we aimed to evaluate the effects of tofacitinib treatment on survival and phenotype in an ALS mouse model. Additionally, we sought to determine the impact of dose and regimen on efficacy.

**Methods:**

*SOD1* ^G93A^ mice, the most used rodent model of ALS, were treated with low- (5 mg/kg) and high-dose (30 mg/kg) tofacitinib following a prevention regimen, an intervention regimen, or a drug-cycling regimen, with survival being the primary outcome. Symptom onset was assessed via body weight, agility, and grip strength measurements. At end-stage disease (i) motor neurons and neuromuscular junctions were counted, (ii) immune populations were quantified via flow cytometry in peripheral blood and spinal cord, (iii) microglial surface marker expression was quantified to assess neuroinflammation, and (iv) bulk RNA-seq was performed on spinal cord.

**Results:**

Low-dose, but not high-dose, tofacitinib significantly increased survival and delayed weight loss. Notably, beginning treatment before symptom onset (prevention) did not offer any survival advantage over the intervention nor cycling regimen; further analyses were pooled by dose. There were no differences in motor neuron or neuromuscular junction counts. Peripheral NK and CD8+ T cells were decreased dose-dependently. Interestingly, spinal cord infiltrating NK cells increased with low-dose tofacitinib, though no other changes in neuroinflammation were observed. RNA-seq revealed that low-dose tofacitinib treatment reversed the dysregulation of multiple immune and metabolic pathways.

**Conclusions:**

These data support the repurposing of tofacitinib as a potential ALS treatment.

## Introduction

Amyotrophic lateral sclerosis (ALS) is a lethal neurodegenerative disease characterized by the destruction of upper and lower motor neurons in the central nervous system (1). Individuals with ALS initially experience muscle weakness with progressive loss in their ability to speak, eat, move, and, eventually, breathe. Death typically occurs 2–4 years from the time of diagnosis. Despite the rapid and ultimately lethal nature of ALS, only a handful of treatments of limited efficacy exist (2), necessitating the development of new treatment options.

The immune system plays a role in ALS pathology in both humans afflicted with the disease and mouse models (3–5). In humans, many immune cell types and pathways in peripheral blood are associated with ALS survival and progression, including monocytes, neutrophils, CD4+ T cells, and natural killer (NK) cells (6–17). Multiple postmortem studies also report corresponding immune infiltration into human ALS spinal cord tissue (10, 18), findings that have been replicated in mouse models (19–21). More recently, we showed that changes in peripheral immune markers occur upstream of clinical changes, reinforcing the idea that the immune system plays an active role in shaping ALS pathology (6). In mice, the role of the immune system in ALS is even more evident, as manipulating specific immune cells can slow or accelerate disease progression (7, 18, 21–27). Together, these data demonstrate that the immune system is a viable therapeutic target in ALS; however, global immune suppression has proven ineffective or accelerated disease, indicating that target-specific immunotherapies against detrimental immune populations and pathways are needed (28–32).

NK cells are a promising target for focused immunotherapy for ALS. These innate immune cells are responsible for destroying cancerous, infected, and damaged cells in the body (33). Typically excluded from the central nervous system, NK cells accumulate in affected regions of the spinal cord and motor cortex in ALS (7, 18), exacerbating neuroinflammation, polarizing microglia to a pro-inflammatory phenotype, and decreasing regulatory T cell (Treg) numbers (18). Moreover, NK cells are cytotoxic to ALS neurons *in vitro* (34), and their depletion extends the lifespan of ALS mice (7). Tofacitinib, an FDA-approved janus kinase (Jak) and signal transducer and activator (STAT) pathway inhibitor, preferentially inhibits Jak1/3 primarily utilized by NK cells (35). We recently demonstrated that tofacitinib can inhibit NK cell interferon-gamma production, suppress cytotoxicity, and decrease overall NK cell levels in the peripheral blood of mice in a dose-dependent manner (34). These findings suggest that suppression of NK cell function by tofacitinib treatment may slow ALS progression.

The aims of this study were to test the efficacy of tofacitinib in mutant *SOD1* ^G93A^ mice, the most used rodent model of ALS. Both male and female mice were included, as we previously showed sex differences in the immune mechanisms of ALS, particularly with NK cells (7). Low (5 mg/kg) and high (30 mg/kg) dosages were examined, as tofacitinib’s effects can be dose-dependent (34). For each dose, three treatment regimens were compared: daily prevention beginning before symptom onset, daily intervention beginning near disease onset, and a cycle beginning before symptom onset consisting of one week on tofacitinib and one week without treatment (36). The cycle regimen was designed to target NK cells while sparing protective Tregs; T cell numbers and function recover more rapidly after tofacitinib is discontinued compared to NK cells, and prolonged tofacitinib treatment disproportionally affects Tregs (37–39). Low-dose, but not high-dose, tofacitinib increased survival and delayed weight loss in ALS mice compared to untreated mice. Notably, the treatment regimen had little effect on survival or symptoms. Peripheral NK cell and CD8+ T cells were reduced dose-dependently. Interestingly, low-dose tofacitinib increased NK cell numbers in the spinal cord, though other sources of neuroinflammation were not increased, and RNA-seq analysis found that tofacitinib reversed changes seen in multiple pathways in ALS spinal cord. Together, these results suggest tofacitinib slows ALS progression via NK cell-independent mechanisms and supports the continued study of tofacitinib and other JAK-STAT inhibitors for treating ALS.

## Materials and methods

### Reagents

Phosphate-buffered saline (PBS), Roswell Park Memorial Institute (RPMI) media, and sodium azide were purchased from Thermo Fisher Scientific (Waltham, MA). Ammonium chloride, potassium bicarbonate, ethylenediaminetetraacetic acid (EDTA), and heat-inactivated fetal bovine serum (FBS) were purchased from Millipore-Sigma (St. Louis, MO). Optimal cutting temperature (O.C.T.) medium was purchased from Sakura (Torrance, CA).

4% Paraformaldehyde (PFA) solution was prepared from powder (Millipore-Sigma) in PBS. Red blood cell lysis buffer consisted of 150 mM ammonium chloride, 10 mM potassium bicarbonate, 0.1 mM EDTA, and 14 mM 4-(2-hydroxyethyl)-1-piperazineethanesulfonic acid (HEPES) (Corning, Corning, NY) in water. Flow cytometry buffer consisted of 2% FBS and 154 µM sodium azide in PBS.

### Mouse model

**Figure 1:**
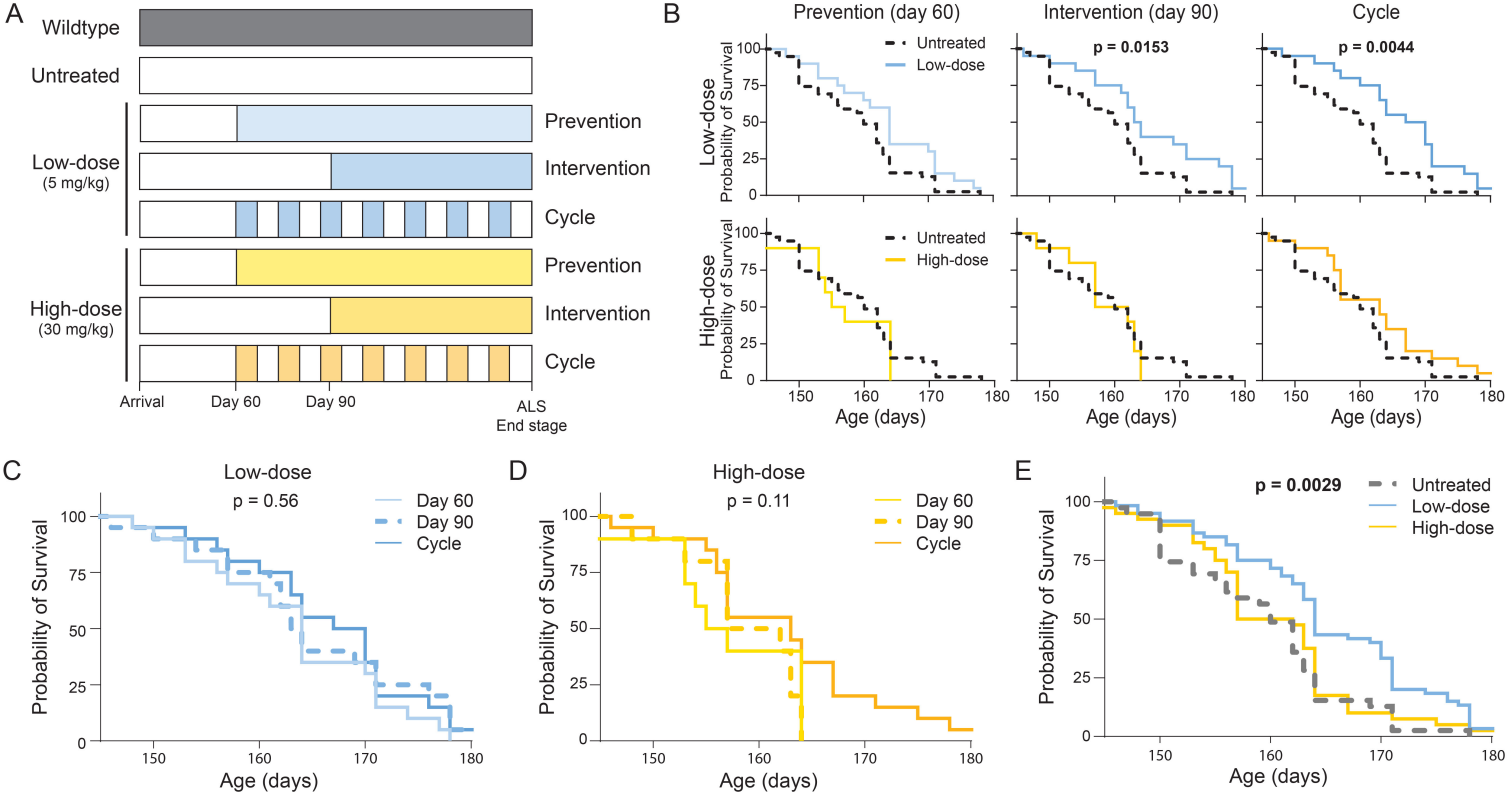
Survival of tofacitinib-treated and untreated *SOD1* ^G93A^ mice. **(A)** *SOD1* ^G93A^ mice were administered tofacitinib through chow formulated to deliver 5 mg/kg (low-dose) or 30 mg/kg (high-dose) daily. Tofacitinib chow was given following three regimens: continuously beginning at 60 days of age (prevention), continuously beginning at 90 days of age (intervention), or weekly cycled regimen beginning at 60 days of age alternating between tofacitinib and normal chow (cycle). Untreated *SOD1* ^G93A^ mice on normal chow served as controls. **(B)** Survival was compared between untreated ALS mice and all six tofacitinib dose-regimen combinations. **(C)** Survival was compared between regimens for low-dose and high-dose tofacitinib. **(D, E)** Survival was compared by tofacitinib dose alone (N = 129, 29–60 mice per group).

Mice for this study were purchased from Jackson Laboratory (Bar Harbor, ME) and included male and female low-copy *SOD1* ^G93A^ ALS mice (B6.Cg-Tg(SOD1*G93A)1Gur/J; Jackson Stock #004435) as well as non-carrier, wild-type (WT) littermates. All mice were housed under specific pathogen-free conditions in a dedicated facility maintained at 20 ± 2°C with a 12/12-hour light/dark cycle. Animals were fed *ad libitum* either AIN-76A rodent diet (WT and untreated ALS mice) or chow containing tofacitinib (Research Diets, New Brunswick, NJ). Three separate cohorts with staggered birth dates were used to ensure reproducibility. Eight different treatment groups were assessed: an untreated ALS group (UT) to serve as a control, three low-dose treated ALS mouse groups (prevention, intervention, cycle; see below and **Figure 1A**), three high-dose treated ALS mouse groups (prevention, intervention, cycle), and an untreated WT group to serve as a reference (**Supplementary Table S1**). Each cohort was composed of both male and female mice, as we have previously observed sex-based differences in immune mechanisms that contribute to ALS progression (7, 10, 19). All mouse studies were performed in accordance with University of Michigan Institutional Animal Care & Use Committee approved protocols (#PRO00010247). Mice were monitored daily by veterinary staff with additional checks by lab personnel at later stages of disease. All studies were conducted in accordance with the United States Public Health Service’s policy on Humane Care and Use of Laboratory Animals.

### Power calculations and randomization

Cohort sizes were determined by doubling cohort sizes from our previous study examining the role of NK cells in ALS, which found a near-significant (p = 0.0615) survival effect (7). Researchers were blinded to drug regimen for immunohistochemistry quantification and immunophenotyping analysis. Half of all animals were used for flow cytometry immunophenotyping and half for RNA-Seq analysis; collection for each type of analysis was alternated in each treatment group with the death of each animal to ensure that samples evenly covered the entire window in which animals reached the terminal endpoint of disease.

### Tofacitinib treatment

Tofacitinib-treated mice received either low-dose (5 mg/kg) or high-dose (30 mg/kg) tofacitinib in specially formulated mouse chow, as previously described (34). Treated mice were placed on one of three treatment schedules: a preventative daily treatment beginning at 60 days of age, an intervention daily treatment beginning at 90 days of age, and a weekly cycled regimen beginning at 60 days of age alternating between tofacitinib and normal chow (**Figure 1A**). All data were generated from at least 2 separate cohorts apart from high-dose prevention and intervention treatments which were discontinued due to lack of efficacy (**Supplementary Table S1**).

### Survival

All ALS mice were allowed to reach the terminal endpoint (*ca*. 160 days) and euthanized when they were no longer able to right themselves after 10 seconds (7). Age-matched WT mice were sacrificed in parallel to provide data at matching timepoints for RNA-seq analysis. Mice were euthanized using a lethal dose of pentobarbital (Vortech Pharmaceutical, Dearborn, MI) followed by exsanguination to ensure euthanasia.

### Physical phenotyping

#### Weight

*SOD1* ^G93A^ and WT mice were weighed weekly until the terminal endpoint of disease.

#### Grip strength

Forelimb grip strength was evaluated biweekly beginning at 35 days of age for each animal using a grip strength meter with a single sensor and a standard pull bar and software (Columbus Instruments, Columbus, OH) (19). For each assessment the peak force (g) was recorded in three consecutive trials of three tests each, averaged, and normalized to body weight (g) obtained the same day to report an average grip strength. Grip strength was not evaluated for cohort 3.

#### Rotarod

Motor function was assessed using a Rotamex-5 rotarod instrument (Columbus Instruments) (7). Mice began training 4 days after arrival, and measurements were performed biweekly beginning at 35 days of age. Mice were acclimated before trials with 5 rpm for 5 min; assessments were performed starting at 0 rpm and increasing by 0.1 rpm/s to a maximum of 30 rpm over 5 min. Time to fall was recorded for 3 trials per mouse with 10-min rest intervals between trials. Values for each trial were averaged to generate a single final value for analysis. Rotarod was not evaluated for cohort 3.

### Sample collection

#### Blood

Following euthanasia, the abdominal and thoracic cavity were opened and blood was collected from the vena cava in a 1 mL syringe (BD Biosciences, Franklin Lakes, NJ), transferred to an EDTA vacutainer (BD Biosciences), and stored on ice until flow cytometry processing (< 1 h).

#### Spinal cord

Following blood collection, the circulatory system was perfused with cold PBS, and the spinal cord from C6 to L6 was excised. C6-T12 was either kept on ice in RPMI until flow cytometry processing (< 1 h) or flash frozen in liquid nitrogen and stored at -80°C until processing for RNA-seq. L1-L6 was placed in PFA for immunohistochemistry.

#### Muscle

Following blood collection and perfusion, the gastrocnemius and tibialis anterior were isolated from the left rear leg, flash frozen in liquid nitrogen, and stored at -80°C until time of analysis.

### Immunohistochemistry

#### Spinal cord

Lumbar spinal cord (L1-L6) segments were processed and analyzed as previously described (19). Briefly, spinal cord segments were fixed in 4% PFA for 24 h then moved through a sucrose gradient before being embedded in O.C.T. medium. Blocks were stored at -80°C until cryostat sectioning, after which 14 µm sections were mounted and stained using standard hematoxylin and eosin (H&E) methods and imaged with an Olympus BX43 microscope (Olympus USA, Center Valley, PA). Motor neurons with an area greater than 300 µm^2^ were counted from 5–6 sections per animal by a blinded observer.

#### Neuromuscular junctions

Muscles were processed and analyzed as previously described (19). Briefly, flash-frozen gastrocnemius and tibialis anterior muscles were embedded in O.C.T. medium and stored at -80°C before preparing 14 µm cryostat sections. Sections were stained for motor endplates, indicated by acetylcholinesterase staining using 5-bromoindoxyl acetate, and for nerve fibers [anti-neurofilament (Millipore-Sigma)], mounted, and imaged with an Olympus BX43 microscope (Olympus USA, Center Valley, PA). NMJs, defined as colocalization of motor endplates and nerve fibers, were counted from 4 sections per muscle per mouse by a blinded observer.

### Flow cytometry

#### Blood

Blood was processed as previously described (19). Briefly, blood was lysed, washed 3 times, and kept in flow cytometry buffer on ice until staining. Cells were counted using a hemacytometer to determine total cell numbers. Cells/µL was calculated by dividing the total cell number with the volume of blood used (µL). Samples were excluded from analysis if they had more than 2x10^5^ cells/µL, 4 times the average white blood cell count for 16-week-old C57BL/6J reported by Jackson Laboratory (Bar Harbor, ME).

#### Spinal cord

Spinal cord was processed as previously described (7, 19). Briefly, isolated thoracic spinal cord (C6-T12) was mechanically and enzymatically dissociated and pressed through a cell strainer. Myelin and debris were removed via Percoll (Cytiva) gradient, and the resulting single cell suspension was washed before staining.

#### Staining and analysis

Cells were treated with Fc blocking antibody (BioLegend, San Diego, CA) for 20 minutes, stained with a panel of antibodies for 30 minutes (**Supplementary Table S2**), washed, and fixed in stabilizing fixative (BD Biosciences) before analysis on a BD Fortessa. Gating was performed as previously described and summarized in **Supplementary Table S2** (7, 19). Microglial size was assessed using forward scatter (FSC), and mean fluorescence intensity (MFI) of CD11c and F4/80 on microglia were normalized to the MFI of microglia stained with a full minus multiple (FMM) panel to account for run-to-run variation. Absolute numbers of cells in blood were calculated using the total cell number and the percentage of each population.

### RNA-seq

#### RNA isolation and sequencing

Following isolation, thoracic (C6-T12) spinal cords were flash frozen in liquid nitrogen and stored at -80°C until RNA extraction. Samples from all cohorts were processed and submitted together after the last cohort was processed to prevent run-to-run variation. Spinal cords were mechanically dissociated before undergoing a TRIzol-chloroform RNA isolation, followed by a clean-up and concentration kit (Norgen Biotek, Thorold, Ontario). Multiplex amplification was used to prepare cDNA with a paired-end read length of 100 bases using an Illumina HiSeq 2000 (Illumina, Inc., San Diego, CA, USA). Library preparation and next generation sequencing were performed by the Advanced Genomics Core at the University of Michigan.

#### RNA-seq data processing

Trimmomatic software was used to remove low-quality reads (Q<30) and sequencing adapters from raw reads (40). The clean reads were subsequently mapped to the mouse reference genome (GRCm38) using HISAT2 (41). The unique mapped reads to mouse genes were summarized with featureCounts (42). Principal component analysis assessed clustering by cohort and sex. If clusters were separated, this was corrected using the ComBat-Seq function from the sva package (V.3.42.0), considering the cohort as a known batch variable (43).

#### Differential gene expression identification

DESeq2 identified differentially expressed genes (DEGs) with a cutoff of p < 0.05 and |log_2_(fold-change)| > 1 (44). Shared and unique DEGs were determined using our in-house gene-set overlap analysis R package, VennDetail (https://github.com/hurlab/VennDetail).

#### Functional enrichment analysis

The functional enrichment analysis of the Kyoto Encyclopedia of Genes and Genomes (KEGG) and gene set enrichment analysis (GSEA) were performed using our in-house richR package (https://github.com/hurlab/richR). Significant pathways were identified with a p-value <0.05 for KEGG enrichment GSEA.

### Collection of RNA from fixed spinal cord tissue

RNA was collected from fixed spinal cord sections using the RNeasy FFPE kit (Qiagen) as per the manufacturer’s instructions. In brief, OCT was thawed and carefully removed from the tissue section. Tissue was minced and washed to remove additional OCT. After washing, the tissue was incubated with PKD buffer and proteinase K, centrifuged, and the supernatant collected. The supernatant was incubated with DNase booster buffer and DNase 1, then buffer and 100% ethanol were added and the supernatant transferred to RNeasy spin columns. RPE buffer was added and columns were spun multiple times to wash the sample before and RNA was eluted using RNase-free H_2_O. Following RNA quantification, samples were frozen at -80° C until time of qRT-PCR analysis.

### Cell culture

Validation studies examining the impact of tofacitinib treatment on NK cell function were performed as previously described using an NK cell line (NK-92, ATCC Cat# CRL-2408) (34). Three concentrations of tofacitinib were used: 15.62 ng/ml (previous *in vitro* concentration), 80 ng/ml (low-dose), and 530 ng/ml (high-dose). Concentrations were calculated based on the mg/kg administered to each mouse divided by the average blood volume (1.5 ml) per mouse. Monocultures of NK-92 cells were cultured overnight with IL-15 stimulation alone (2.33 nM, PeproTech) or with IL-15 stimulation in conjunction with tofacitinib treatment, then cells were collected, washed, and frozen at -80° C until time of analysis. Co-cultures with K-562 cancer cells (ATCC Cat# CCL-243) were conducted at a 10:1 ratio for four hours with IL-15 stimulation alone or with tofacitinib treatment. Following co-culture, cells were collected, washed and RNA isolated using the RNeasy isolation kit (Qiagen) and frozen at -80° C until time of analysis.

### Quantitative real-time PCR

Gene expression in NK-92 cell pellets and spinal cord tissue sections was quantitated using qRT-PCR as previously described (34). In brief, cDNA was first generated using iScript Reverse Transcription Supermix (Bio-Rad) as per the manufacturer’s instructions, run in a ProFlex PCR Thermal Cycler (Applied Biosystems). Next, qPCR reactions were run in triplicate in a StepOnePlus System (Applied Biosystems) using TaqMan Universal PCR Master Mix (Applied Biosystems). For NK-92 cells, probes for IL-10 (Hs00961622_m1), TNF-α (Hs00174128_m1), and IFN-γ (Hs99999041_m1) were used, multiplexed with β-actin (Hs01060665_g1) serving as a control. C_T_ values were used to calculate ΔC_T_ and ΔΔC_T_ using the housekeeping gene and IL-15-alone samples. For spinal cord samples, probes for Btnl10 (Mm00507067_m1), Ctsg (Mm00456011_m1), and Elane (Mm00469310_m1) were used, with Ywhaz (Mm03950126_s1) serving as a control. C_T_ values were used to calculate ΔC_T_ and ΔΔC_T_ using the housekeeping gene and WT samples.

### Western blots

Western blot analysis was performed as previously described to examine STAT3 and STAT5 phosphorylation (34). In brief, cell pellets were lysed in RIPA buffer (Thermo Fisher Scientific), and protein was isolated. Blots were prepared via SDS-PAGE in 10% acrylamide gels, transferred to Immobilon-FL PDVF membranes (Millipore), and immunoblotted with Phospho-STAT3 antibody (Cell Signaling Technology, Cat# 9145) or Phospho-STAT5 antibody (AbCam, Cat# ab32364). Anti-tubulin (Abcam, Cat #ab6160) was used as a control.”

### Statistics

#### Basic statistics

Data were assessed for normality by Shapiro-Wilk test. Three or more groups at the endpoint of disease with normally distributed data were analyzed by ANOVA and a *post-hoc* Tukey’s test was performed for significant comparisons. Three or more groups with non-normally distributed data were analyzed by Kruskal-Wallis and a *post-hoc* Dunn’s test was performed for significant comparisons with p-values corrected for multiple comparisons using Bonferroni correction.

#### Controlling for cohort effects

Data were assessed to determine if cohorts were a significant confounding factor. Untreated ALS mice were used to identify differences between cohorts, and corrections were then applied to all mouse groups (**Supplementary Figure S1**). A significant cohort effect was observed for body weight, motor neuron count, and immune markers in the CNS, including CD4+ T cells, neutrophils, microglial size (FSC), and microglial CD11c expression. Body weight linear mixed model analysis was adjusted for cohort variation. Motor neuron count, spinal CD4+ T cells, and spinal neutrophils were normalized by dividing values by the average values for untreated mice in that cohort, then multiplying by the average of all untreated mice. Microglial FSC and CD11c were normalized by dividing values by the average values for untreated mice in that cohort.

#### Survival

Survival curves for each treatment group were generated using Kaplan-Meier methods and significance was assessed using a log-rank test.

#### Physical phenotyping

Segmented linear mixed models were used to determine the inflection point when loss of function began or accelerated for each group of mice, including loss of weight, grip strength, and agility. Body weight percentage was calculated using the body weight at 60 days of age as baseline.

#### Covariates

Where appropriate, the cohort of the mice was used as a covariate.

## Results

### Low-dose tofacitinib extends survival in ALS mice

To test whether tofacitinib can slow the rate of ALS progression and reduce neuroinflammation, we treated *SOD1* ^G93A^ ALS mice orally with tofacitinib pressed into chow at low (5 mg/kg) or high (30 mg/kg) doses, which we compared to untreated *SOD1* ^G93A^ mice. We tested 3 separate treatment regimens for each tofacitinib dose: a preventative model starting at 60 days of age before symptom onset, an intervention model starting at 90 days of age following initial symptom onset, and an intermittent “cycle” regimen where mice were placed on tofacitinib chow or control chow on alternate weeks starting at 60 days of age (**Figure 1A**).

First, we examined the impact of tofacitinib dose and regimen on ALS survival. Low-dose tofacitinib significantly extended survival of ALS mice compared to untreated control ALS mice in the intervention (6.2 day increase; p = 0.02) and cycle (7.3 day increase, p = 0.004) regimens and showed a trend towards improvement in the prevention regimen (p = 0.06; **Figure 1B**). In contrast, no high-dose tofacitinib regimen increased ALS mouse survival, with prevention and intervention paradigms trending towards a decrease in survival. Consequently, based on the findings in the first cohort, high-dose prevention and intervention treatment groups were discontinued for the remainder of the study. Slight differences were observed between male and female mice, though patterns were consistent throughout (**Supplementary Figure S2**). Direct comparisons of treatment regimens did not find differences for either low- or high-dose tofacitinib (**Figures 1C, D**). Thus, subsequent analyses pooled dosage groups to increase power and facilitate interpretation. Results were consistent in this combined analysis: low-dose, but not high-dose, tofacitinib significantly increased survival (**Figure 1E**). Together, these data suggest that low-dose tofacitinib improves ALS survival.

### Tofacitinib delays weight loss but does not improve end-stage motor neuron or NMJ counts

**Figure 2:**
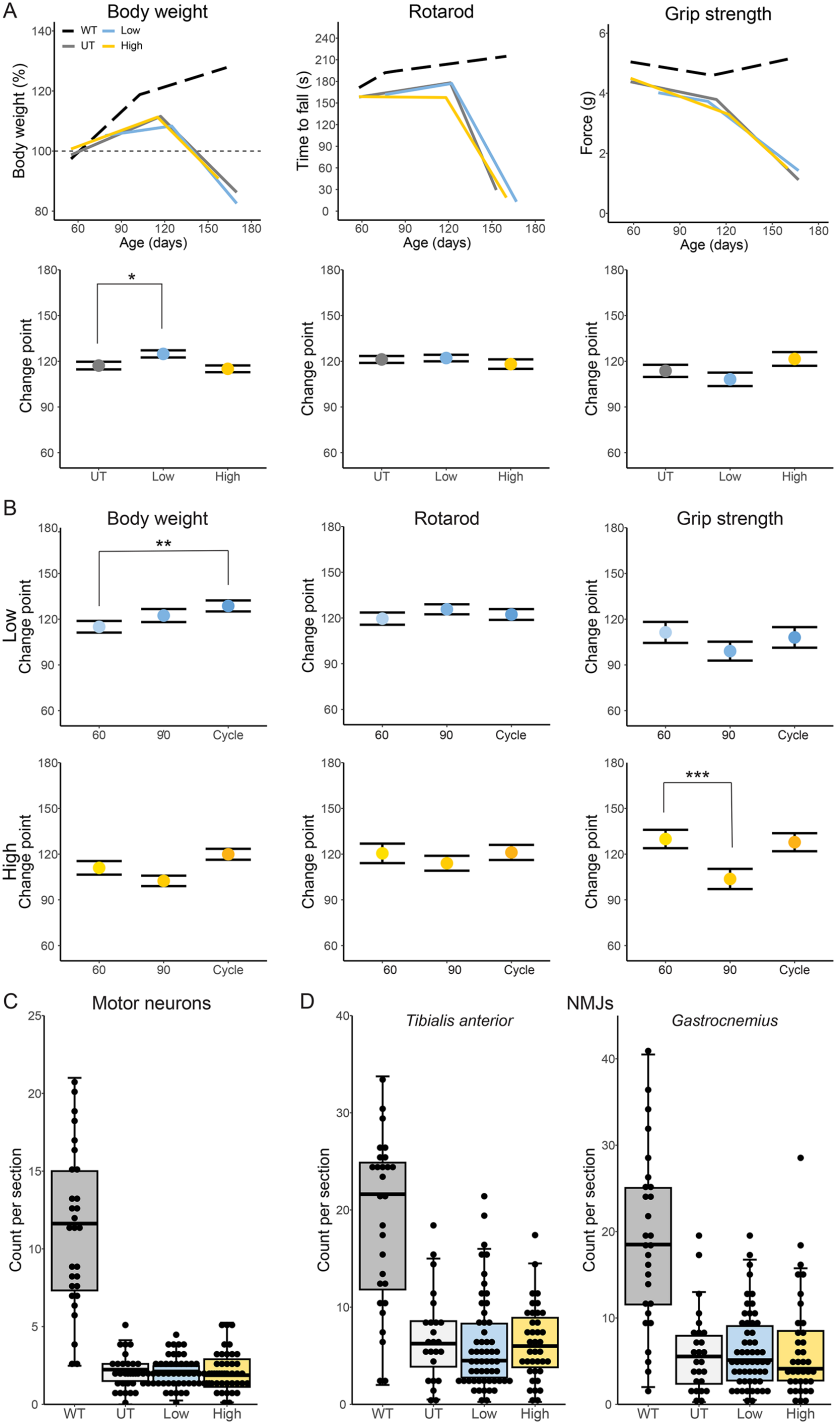
Physical phenotyping of tofacitinib-treated and untreated *SOD1* ^G93A^ mice. **(A, B)** Body weight, grip strength, and rotarod phenotyping was performed every 2 weeks beginning at 60 days of age and analyzed via linear mixed model for change point **(A)** by dose and **(B)** by regimen. Body weight is presented as percentage of body weight at 60 days of age and is normalized by cohort. Grip strength was normalized to weight (N = 129; 10–60 mice per group). **(C)** Motor neurons were counted in stained lumbar spinal cord sections (N = 122; 26–58 mice per group). **(D)** Neuromuscular junctions were counted in sections of the tibialis anterior (N = 118; 24–56 mice per group) and gastrocnemius muscles (N = 120; 26–56 mice per group). WT mice included for reference only and are excluded from statistical analysis. *p < 0.05, **p < .01, and ***p < .001, by linear mixed models **(A, B)**, Kruskal-Wallis **(C)**, or one-way ANOVA **(D)**.

Next, we explored whether tofacitinib improves physical phenotypes during ALS, assessed via change point in a linear mixed-model analysis of the decline in body weight, grip strength, and agility, as well as the end-stage motor neuron and NMJ counts. Low-dose tofacitinib significantly delayed the onset of weight loss (7.6 days) in ALS mice but did not impact agility or grip strength (**Figure 2A**). Regimen had a moderate effect, with the onset of weight loss significantly delayed in mice on the low-dose cycle regimen and slightly, but not significantly, hastened with high-dose intervention (**Figure 2B**, **Supplementary Figure S3A**). Interestingly, male, but not female, mice receiving high-dose tofacitinib on the prevention or cycling regimens had a significant delay in grip strength decline inflection point not seen with low-doses (**Supplementary Figures S3A-C**). Tofacitinib did not affect the number of motor neurons in lumbar spinal cord sections (**Figure 2C**) nor NMJ counts in the tibialis anterior or gastrocnemius (**Figure 2D**). These data suggest that tofacitinib can delay disease onset but does not result in improvement of end-stage motor neuron or NMJ counts.

### Tofacitinib affects peripheral and infiltrating immune populations without any change in neuroinflammation

**Figure 3:**
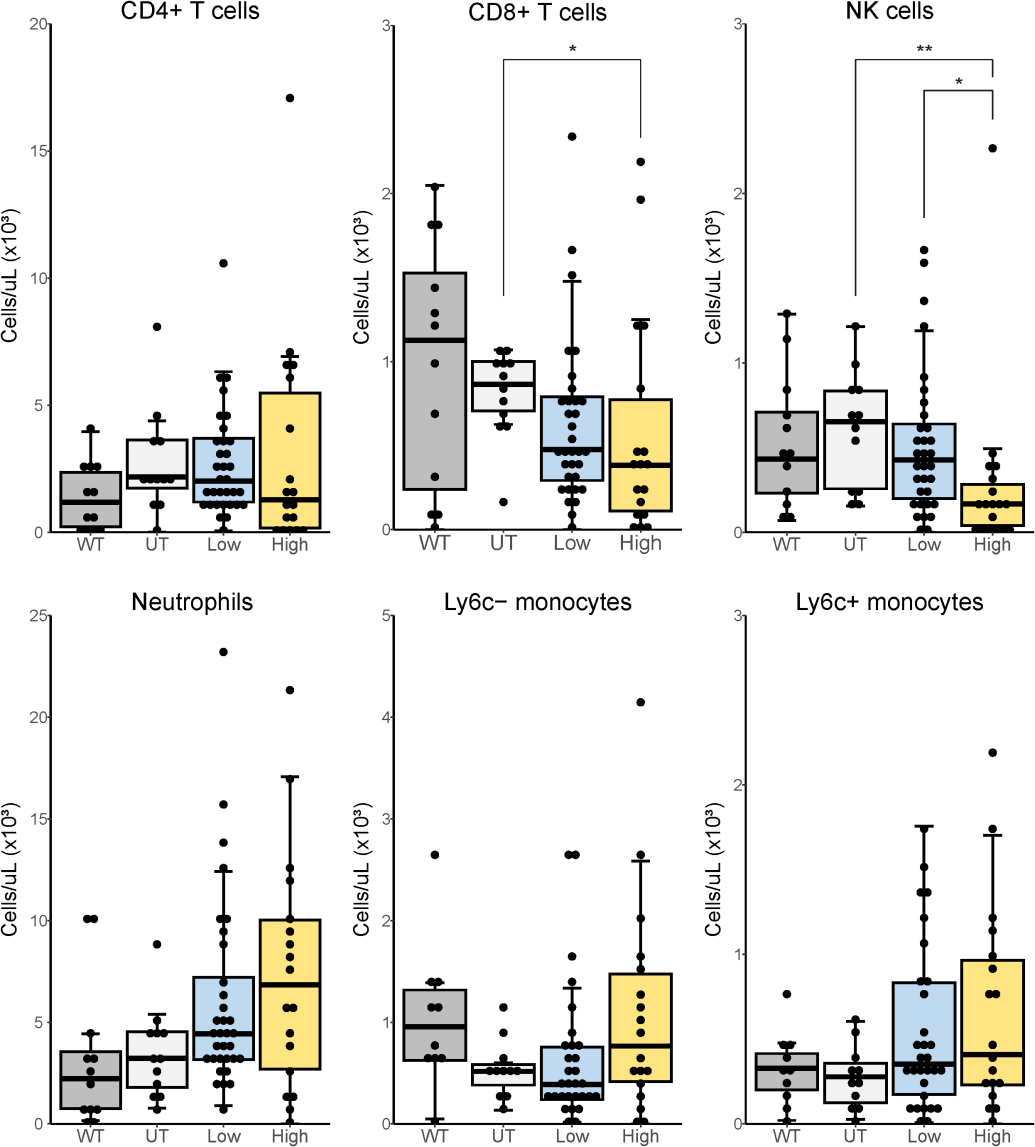
Immune phenotyping in peripheral blood of tofacitinib-treated and untreated *SOD1* ^G93A^ mice. *SOD1* ^G93A^ mice were sacrificed at end-stage disease with age-matched WT mice. Peripheral blood immune cell populations were analyzed via flow cytometry, as indicated. N = 63, 12–30 mice per group (CD4+ T cells, CD8+ T cells, NK cells, neutrophils); N = 59, 10–20 mice per group (Ly6c- and Ly6c+ monocytes). WT mice included for reference only and are excluded from statistical analysis. *p < 0.05, **p < 0.01, by Kruskal-Wallis with *post-hoc* Dunn’s test with Bonferroni correction.

To assess the effect of tofacitinib treatment on immune populations and inflammation we quantified immune cell numbers by flow cytometry in peripheral blood and thoracic spinal cord at the terminal endpoint of disease. Tofacitinib significantly reduced the total number of NK cells in peripheral blood in a dose-dependent manner (**Figure 3**). CD8+ T cells were similarly affected in a dose-dependent manner, though the reduction of CD8+ T cells with low-dose tofacitinib did not reach significance. Few differences were observed when stratified by sex or regimen (**Supplementary Figure S4**).

**Figure 4:**
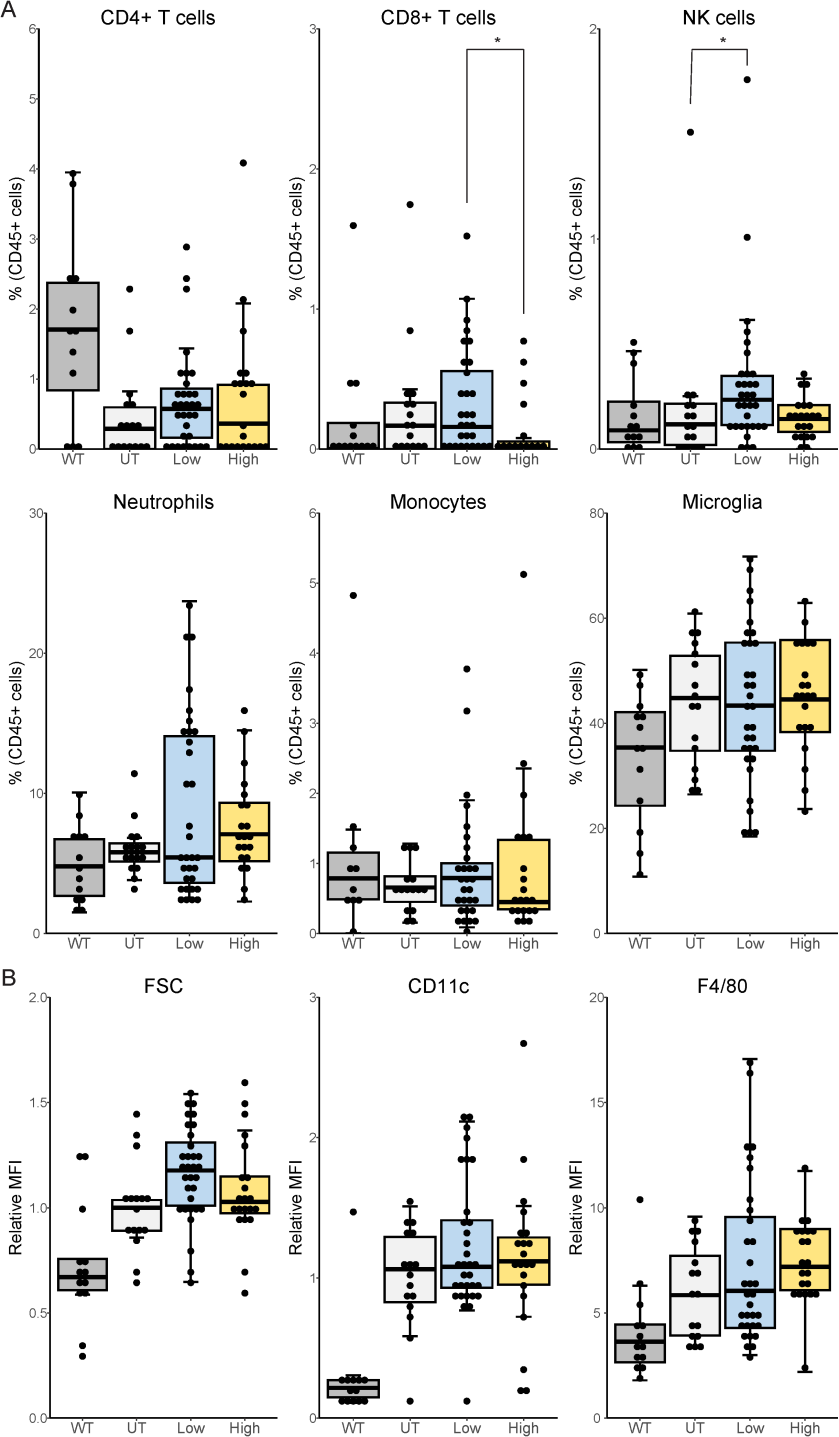
Immune phenotyping in the spinal cord of tofacitinib-treated and untreated *SOD1* ^G93A^ mice. *SOD1* ^G93A^ mice were sacrificed at end-stage disease and flow cytometry analyzed spinal cord immune cell populations. **(A)** Immune populations in the spinal cord. **(B)** Microglia phenotype markers. CD4+ T cells and neutrophils were normalized by cohort **(A)**, as were forward scatter (FSC) and CD11c **(B)**. N = 69, 13–32 mice per group. WT mice included for reference only and are excluded from statistical analysis. *p < 0.05, by Kruskal-Wallis with *post-hoc* Dunn’s test with Bonferroni correction or by one-way ANOVA with *post-hoc* Tukey test as indicated by a Shapiro-Wilk test using p=0.05.

Given its effects on survival and peripheral immune populations, we next sought to determine if tofacitinib altered neuroinflammation in the spinal cord of ALS mice. Surprisingly, low-dose tofacitinib increased the proportion of NK cells in the spinal cord compared to untreated ALS mice (**Figure 4A**). There were no other differences in proportions of infiltrating immune cells or microglia, and no effect of sex or regimen was observed (**Supplementary Figure S5**). Microglial activation was also evaluated, and tofacitinib did not affect microglial size – assessed using FSC – or surface expression of activation markers CD11c and F4/80 (**Figure 4B**). The impact of these doses on NK cell function was validated *in vitro* using an NK cell line to assess expression of genes for IFN-γ, TNF-α, and IL-10 (34). Interestingly, we found that high-dose tofacitinib increased cytokine expression in NK cells, though this effect did not reach statistical significance (**Supplementary Figure 6A**). The impact of tofacitinib on NK cell STAT phosphorylation was also examined using Western blot and showed that increased dosages of tofacitinib did not adversely impact other members of the Stat family at higher doses (**Supplementary Figure 6B**). Together, these results suggest that tofacitinib treatment suppresses peripheral NK cell and CD8 T cell levels but does not significantly decrease immune accumulation or microglial activation in the spinal cord.

### Tofacitinib alters regulation of immune and metabolic pathways in the spinal cord

**Figure 5:**
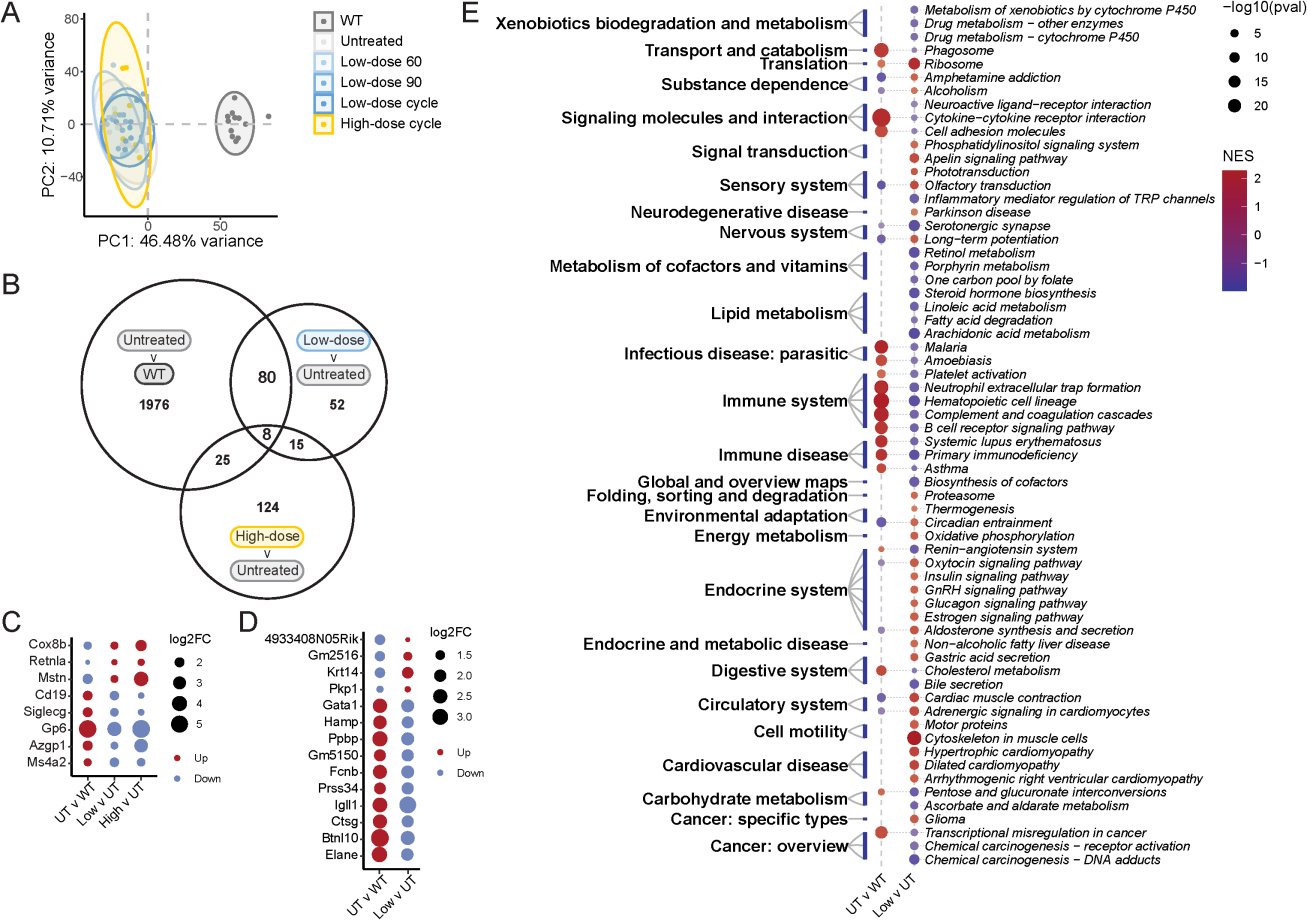
Transcriptomics analysis of spinal cord from tofacitinib-treated and untreated *SOD1* ^G93A^ mice. **(A)** Principal component analysis; color indicates treatment group after correction by cohort. **(B)** Comparisons of overlapping and unique differentially expressed genes (DEGs) across all three comparisons – untreated ALS versus untreated wild-type (UT v WT), low-dose tofacitinib versus untreated ALS mice (Low v UT), and high-dose tofacitinib versus untreated ALS mice (High v UT). **(C)** Expression levels of eight DEGs shared by all three comparisons. **(D)** Expression levels of top 10 DEGs each for untreated ALS versus untreated WT and low-dose tofacitinib versus untreated ALS by log_2_(fold-change) (log_2_FC). See **Supplementary Table S3** for the complete list of DEGs. **(E)** Gene set enrichment analysis of untreated ALS versus untreated WT and low-dose tofacitinib versus untreated ALS. See **Supplementary Table S4** for the complete list of GSEA results. A cutoff of p < 0.05 and |log_2_(fold-change)| > 1 was used to identify DEGs. N = 58, 8–12 mice per group **(A)**, 8–28 mice per group **(B-E)**.

Finally, we examined gene expression in thoracic spinal cord using bulk RNA-seq to identify which pathways were altered by tofacitinib treatment. Untreated WT littermates served as a reference to identify ALS-dysregulated pathways and determine which pathways were improved by tofacitinib treatment. We first performed principal component analysis to assess significant gene expression differences that resulted from cohort, sex, or dose-regimen combination. Different clusters were observed when comparing cohorts, which were batched-corrected (**Supplementary Figure S7**). Unsurprisingly, genotype, i.e., WT vs *SOD1* ^G93A^, most strongly influenced clustering contributing to over 40% of variation (**Figure 5A**). Within ALS mice, neither treatment nor sex profoundly separated clusters.

DEG analysis was then performed for 3 comparisons: untreated ALS versus untreated WT (to identify ALS-dysregulated DEGs), low-dose tofacitinib versus untreated ALS mice (to identify ALS-dysregulated DEGs that low-dose tofacitinib reverses), and high-dose tofacitinib versus untreated ALS mice (to identify ALS-dysregulated DEGs that high-dose tofacitinib reverses). We identified over 2,000 DEGs in untreated ALS versus WT mice, 155 DEGs in low-dose versus untreated ALS mice and 172 DEGs in high-dose versus untreated ALS mice (**Figure 5B**, **Supplementary Table S3**). Of these, there were eight DEGs shared by all three comparisons, including *Cox8b*, *Mstn*, *Azgp1*, and *Ms4a2* (**Figure 5C**).

Our subsequent analysis focused on DEGs and pathways that were reversed by low-dose tofacitinib, as high-dose treatment did not improve survival. We found that 80 DEGs were reversed by low-dose treatment but not high-dose treatment, including *Gata1*, *Fcnb*, *Ctsg*, *Btnl10*, and *Elane* (**Figure 5D**). These findings were validated by isolating RNA from fixed spinal cord tissue and using qRT-PCR to quantify *btnl10*, *ctsg*, *elane* expression. Consistent with the RNA-Seq findings, gene expression for these three genes was increased in untreated ALS mice but reversed by low-dose tofacitinib treatment (**Supplementary Figure S8**). Pathway analysis by GSEA identified ALS-dysregulated pathways reversed by low-dose tofacitinib treatment. In untreated ALS spinal cords, multiple pathways associated with inflammation were upregulated compared to WT mice, and tofacitinib reversed many of these dysregulated pathways, including those associated with cytokine-cytokine receptor interaction, platelet activation, neutrophil extracellular trap formation, and complement and coagulation cascades (**Figure 5E**, **Supplementary Table S4**). Tofacitinib also impacted numerous other pathways that were not dysregulated in ALS versus WT, including metabolism of cofactors and vitamins, lipid metabolism, the endocrine system, and cell motility. Together, these findings indicate that tofacitinib downregulates neuroinflammatory pathways dysregulated in ALS, and affects metabolic pathways not dysregulated in the spinal cord in untreated ALS.

## Discussion

The immune system contributes to ALS progression (3, 4), with NK cells playing a key role (6, 7). We previously demonstrated that tofacitinib, a JAK/STAT inhibitor (45–47), suppresses overall NK cell numbers, cytotoxicity, and cytokine production *in vitro*, *ex vivo*, and *in vivo* (34). Therefore, to determine whether tofacitinib has therapeutic potential for treating ALS, we treated *SOD1* ^G93A^ mice with low-dose (5 mg/kg) or high-dose (30 mg/kg) tofacitinib using multiple dosing regimens. Low-dose tofacitinib treatment significantly prolonged survival and delayed the onset of weight loss, while high-dose treatment had no effect or exacerbated disease. Initiating tofacitinib after symptom onset did not drastically differ from initiating treatment before symptom onset, a particularly promising finding since patients often face years-long delays between ALS disease onset and diagnosis (1). Despite these salutary benefits, tofacitinib did not perceptibly alter motor neuron or NMJ number at disease end-stage, and there was no drug-mediated improvement in grip strength or agility. Tofacitinib decreased peripheral NK cells in a dose-dependent manner, and high-dose tofacitinib also significantly decreased peripheral CD8+ T cells. Interestingly, low-dose tofacitinib increased spinal cord infiltration of NK cells, though no increase in microglial activation or other markers of neuroinflammation were observed. Transcriptomics analysis of spinal cord at end-stage disease found that low-dose tofacitinib reversed several ALS-dysregulated DEGs primarily linked to immune pathways and additionally influenced metabolic pathways unrelated to ALS.

Interestingly, the tofacitinib regimen used did not have a significant effect on the response to treatment. No differences in survival or immune populations and only slight differences in phenotype were observed between mice on prevention or intervention regimens for both low- and high-doses. One potential explanation is that NK cells and CD8+ T cells do not play major protective roles at any point in ALS and, thus, there may be limited advantage with earlier tofacitinib treatment (3, 4, 48). Another possibility is that the use of the intervention regimen beginning at 90 days of age preceded inflammatory changes in ALS, as the immune system shifts from an anti-inflammatory phenotype to a pro-inflammatory phenotype between 90 and 120 days of age in mice (19), after both prevention and intervention regimens were initiated.

Despite an increase in survival and delay in weight-loss onset, the only significant change in immune populations in the spinal cord with tofacitinib treatment was a slight increase in infiltrating NK cells, counter to our hypothesis that infiltrating NK cells are detrimental in ALS. One possible explanation is that NK cell function is more important than NK cell number; we have found that NK cell expression of NKp46, NKp30, and NKG2D, as well as CD62L+ and CD27+ subpopulations, are better predictors of ALS progression than the number of NK cells alone (6). Others have found that tofacitinib has a greater impact on NK cell phenotype, such as cytotoxicity, than on viability (49). This potential decrease in NK cell activity is supported by the lack of subsequent microglial activation, though *in vivo* NK cell function was not tested in the current study. Indeed, specific NK cell subpopulations may play a role in disease: in our own studies we have found significant dysregulation of NK cells in individuals with ALS Previous studies have shown that CD11b and CD27 expression on human NK cells identify subpopulations with increased ability to differentiate, express cytokines, or lyse target cells depending on their surface expression (50). Moreover, a recent report suggested that specific NK cell subpopulations may contribute to disease progression in individuals with ALS (51). Indeed, at least one study reported that NK cells can drive ALS via the release of IFN-γ inside the CNS which in turn drives activation of resident Immune cells, particularly the microglia (18). These findings suggest that the NK cells accumulating in the CNS of low-dose mice may have altered functionality, particularly reduced release of IFN-γ, though this was not tested in the current study.

The unexpected finding that low-dose tofacitinib extended survival, while high-dose tofacitinib may exacerbate disease, may be due to a number of factors. First, tofacitinib may adversely impact CD4+ T cell function, including Th2 cells and Tregs which have a net protective effect particularly in early stages of disease (12, 26). As ALS progresses, the balance shifts towards Th1 T cells, which drive disease through microglial polarization and Treg inhibition (52, 53). Tofacitinib exerts differing effects on CD4+ T cells based on dose: low-dose tofacitinib impacts effector T cell function, including Th1 polarization, while higher doses may adversely impact the function of protective Tregs (39, 54–56). Thus, a reduction of Treg function early in disease may explain why the high-dose prevention regimen was associated with reduced survival, though T cell function and polarization was not tested in the current study. With regards to NK cells, we found that higher concentrations of tofacitinib were associated with increased cytokine production, though these changes were not statistically significant. Nonetheless, this may explain why mice receiving high-dose regimens saw reduced survival rates. While tofacitinib primarily inhibits Jak1/3 it can affect the activity of other members of the Jak family (57), so it is possible that higher doses adversely impact associated pathways in NK cells, other immune cells, or other cell types such as neurons. Though we observed no off-target effects in NK cells culture *in vitro*, other cell types were not tested. In addition, mice receiving high doses tofacitinib experienced a trend towards more rapid weight loss. Clearance of the drug, particularly at high doses in female mice, may also have played a role. Our previous pharmacokinetic analyses showed increased maximal concentration in the blood of female mice following drug administration as well reduced drug clearance (34); this may explain why female mice in particular benefited from the use of drug cycles. Other off-target tofacitinib effects, including increased toxicity, likely did not contribute to reduced survival in the high-dose regimens, as tofacitinib is routinely prescribed at higher doses with minimal increase in adverse events (58, 59), and we did not previously observe any signs of toxicity or infection in low-dose or high-dose mice (34). However, it is also unclear whether prolonged use of tofacitinib is toxic to neurons: tofacitinib is somewhat able to cross the blood-brain barrier (60), has shown mixed results in animal models of multiple sclerosis (61, 62), and has caused demyelination in at least one case report (63). Therefore, it is possible that higher doses of tofacitinib may be toxic to motor neurons through heretofore unexplored mechanisms.

Transcriptomics analysis of the spinal cord revealed that tofacitinib reversed pathways dysregulated during ALS. These pathways were primarily related to inflammation, but metabolic pathways were impacted as well. Tofacitinib reversed the most upregulated pathways in ALS, including pathways associated with hematopoietic cell lineage, neutrophil extracellular trap formation, and cytokine-cytokine receptor interactions. Interestingly, tofacitinib treatment also resulted in the downregulation of multiple metabolic pathways that were not dysregulated in untreated ALS mice. These included pathways associated with lipid metabolism for steroid hormone biosynthesis, linoleic acid metabolism, arachidonic acid metabolism, and fatty acid degradation. Tofacitinib’s association with altered lipid metabolism is well-established, primarily observed as increases in LDL and HDL cholesterol in multiple studies of patients on tofacitinib (64, 65). Additionally, tofacitinib has been shown to reduce cachexia in rodent arthritis models (66, 67), and is associated with weight gain in humans (68, 69). This is particularly promising given that ALS has long been associated with metabolism alterations observed in plasma (70, 71), and dysregulation of lipid metabolism is a top predictor of disease progression (72).

There are limitations to this study. Tofacitinib blood levels were not assessed, and differences in the ease and ability for ALS mice to reach the chow may have affected dosing at later stages of disease. We have also previously found sex differences in tofacitinib clearance rates (34). The impact of tofacitinib was not assessed in other mouse models of ALS. *SOD1* ^G93A^ mice, as well as ALS patients harboring *SOD1* mutations, do not develop the TDP-43 proteinopathy seen in 95% of ALS patients (73, 74). Thus, additional experiments using alternate mouse models, such as those based on *C9orf72* expansions, may be warranted. Mouse models also present issues in both the lack of upper motor neuron disease and the limited amount of tissue. For this reason, motor neurons were counted in the lumbar spinal cord, and the thoracic spinal cord was used for flow cytometry and transcriptomics. Moreover, while specific NK cell subpopulations may contribute to ALS progression (51), NK cell subpopulations were not characterized in this study. In addition, clinical trials could benefit from a better understanding of the role of NK cells and NK cell subpopulations in ALS. Preclinical studies including adoptive transfer of specific subpopulations of NK cells or NK cells pre-treated them with tofacitinib could further elucidate the mechanisms by which low-dose tofacitinib slow disease progression. Finally, it must be acknowledged that few ALS treatments have translated from mouse to man (73, 74). However, it is likely that tofacitinib will be effective in human ALS as well, as similar dysregulation of Jak3 is seen in both mouse and man (75). Tofacitinib preferentially inhibits Jak1/3; however, other jakinibs, such as baricitinib, may also warrant study as they differ slightly in Jak-specificity and blood-brain barrier penetrability (47, 76–78).

## Conclusion

There is great potential for tofacitinib as an ALS therapeutic. Further preclinical studies, particularly those in ALS mouse models that develop TDP-43 proteinopathy or studies designed for increased sensitivity for symptom onset and progression, may be helpful in guiding clinical translation. However, these studies face diminishing returns due to the poor predictive power of mouse studies for human translation. Additionally, there is already a significant body of research on tofacitinib in humans, including studies on dose-effect, long-term use, and the impact of tofacitinib on other immune cell types including monocytes, T cells, and B cells, facilitating dosing in individuals with ALS and translation to the clinic (37, 58, 79). Lastly, tofacitinib is not predicted to interact with either riluzole or edaravone, the two most commonly prescribed ALS medications (80, 81). Based on those factors and the findings presented here, tofacitinib is a promising therapeutic candidate for ALS and is ready for translation into clinical trials.

## Data Availability

The original bulk RNA-Seq data presented in this study can be found at ArrayExpress (https://www.ebi.ac.uk/biostudies/arrayexpress), accession number E-MTAB-16025. Differentially expressed gene (DEG) data and gene set enrichment analysis (GSEA) data are available in **Supplementary Materials**. All other original data will be made available by the authors upon request.
