# Supplementary material for: Tofacitinib extends survival in a mouse model of ALS through NK cell-independent mechanisms
Source: Front Immunol. 2025 Nov 5;16:1662197. doi: 10.3389/fimmu.2025.1662197 (PMC12626996; doi:10.3389/fimmu.2025.1662197)
Supplement: Supplementary file 3 [file DataSheet1.docx]

**Table S1. Count of mice by cohort and treatment.**

| ​ | ​ | ​ | **Low-dose**​ **tofacitinib** | | | **High-dose**​ **tofacitinib** | | |
| --- | --- | --- | --- | --- | --- | --- | --- | --- |
|  | **Wild-type** | ***SOD1*^G93A^** | ***SOD1*^G93A^** | | | ***SOD1*^G93A^** | | |
| ​ | ​ **Untreated** | **Untreated**​ | **Day 60**​ | **Day 90**​ | **Cycle**​ | **Day 60**​ | **Day 90**​ | **Cycle**​ |
| Cohort 1​ | | | | | | | | |
| Male​ | 5​ | 5​ | ​ | ​ | ​ | 5​ | 5​ | 5​ |
| Female​ | 5​ | 5​ | ​ | ​ | ​ | 5​ | 5​ | 5​ |
| Cohort 2​ | | | | | | | | |
| Male​ | 5​ | 5​ | 5​ | 5​ | 5​ | ​ | ​ | ​ |
| Female​ | 5​ | 5​ | 5​ | 5​ | 5​ | ​ | ​ | ​ |
| Cohort 3​ | | | | | | | | |
| Male​ | 5​ | 5​ | 5​ | 5​ | 5​ | ​ | ​ | 5​ |
| Female​ | 5​ | 5​ | 5​ | 5​ | 5​ | ​ | ​ | 5​ |
| **Total​** | **30​** | **30​** | **20​** | **20​** | **20​** | **10​** | **10​** | **20​** |

**Table S2. Flow cytometric staining panels and cell populations.** All antibodies were purchased from Biolegend. Mean fluorescence intensity (MFI) for microglial markers CD11c and F4/80 were normalized to MFI from full minus multiple (FMM) control panel.

| **Panel** | **Tissue** | **Antibodies (Catalog #)** | **Analyzed cell populations** |
| --- | --- | --- | --- |
| Lymphoid Control | Blood & spinal cord | BV421 IgG (400157)  FITC CD3 (100203)  PE IgG (400211)  PerCP-Cy5.5 IgG (400150)  APC CD45 (147707)  PE-Cy7 IgG (400522)  APC-Cy7 IgG (400623) |  |
| Lymphoid | Blood & spinal cord | BV421 CD8 (100738)  FITC CD3 (100203)  PE NK1.1 (108708)  PerCP-Cy5.5 CD19 (115532)  APC CD45 (147707)  PE-Cy7 CD49b (108921)  APC-Cy7 CD4 (100413) | CD4+ T cells (CD45+ CD3+ CD19- CD4+ CD8-)  CD8+ T cells (CD45+ CD3+ CD19- CD4- CD8+)  NK cells (CD45+ CD3- CD19- NK1.1+ CD49b+) |
| Myeloid | Blood | BV421 CD11c (117330)  FITC Ly6c (128006)  PE F4/80 (123110)  PerCP-Cy5.5 CD3 (100326)  PerCP-Cy5.5 CD19 (115532)  APC CD45 (147707)  PE-Cy7 Ly6g (127617)  APC-Cy7 CD11b (101226) | Neutrophils (CD45+ CD3- CD19- CD11b+ Ly6g+)  Ly6c+ monocytes (CD45+ CD3- CD19- CD11b+ Ly6g- Ly6c+)  Ly6c- monocytes (CD45+ CD3- CD19- CD11b+ Ly6g- Ly6c-) |
| Myeloid Control | Spinal cord | BV421 IgG (400157)  FITC Ly6c (128006)  PE IgG (400211)  PerCP-Cy5.5 CD3 (100326)  PerCP-Cy5.5 CD19 (115532)  APC CD45 (147707)  PE-Cy7 Ly6g (127617)  APC-Cy7 CD11b (101226) |  |
| Myeloid | Spinal cord | BV421 CD11c (117330)  FITC Ly6c (128006)  PE F4/80 (123110)  PerCP-Cy5.5 CD3 (100326)  PerCP-Cy5.5 CD19 (115532)  APC CD45 (147707)  PE-Cy7 Ly6g (127617)  APC-Cy7 CD11b (101226) | Neutrophils (CD45+ CD3- CD19- CD11b+ Ly6g+)  Ly6c+ monocytes (CD45^high^  CD3- CD19- Ly6g- CD11b+  Ly6c+)  Microglia (CD45^mid^ CD3- CD19- Ly6g- CD11b+)  Surface markers (CD11c, F4/80) |

**Table S3. List of differentially expressed genes (DEGs) by comparison.**

See excel file

**Table S4. Complete GSEA results for untreated versus wild-type and low-dose versus untreated comparisons.**

See excel file

**Supplementary Figure S1. Examination of cohort effect by comparing untreated *SOD1*^G93A^ mice.** Untreated *SOD1*^G93A^ mice were grouped by cohort and endpoint data was assessed for significant differences. **(A)** Kaplan-Meier survival curves. **(B)** Counts of motor neurons in the spinal cord and neuromuscular junctions in the tibialis anterior and gastrocnemius muscles. **(C)** Absolute cell counts of immune populations in peripheral blood assessed by flow cytometry. **(D)** Immune populations in the spinal cord assessed via by cytometry. **(E)** Microglial activation marker expression. *p < 0.05 by one-way ANOVA (MN, CNS neutrophils, FSC, CD11c) or Kruskal-Wallis test (CNS CD4+ T cells) as indicated by a Shapiro-Wilk test using p=0.05. N = 29, 4-10 mice per group.

**Supplementary Figure S2. Survival of tofacitinib-treated and untreated *SOD1*^G93A^ mice stratified by sex.** Survival was compared between untreated mice versus all six tofacitinib dose-regime combinations (**Fig 1A**) and by tofacitinib dose alone in **(A)** male (N = 65, 5-30 mice per group) and **(B)** female mice (N = 64, 5-30 mice per group). *p < 0.05, by log-rank test.

**Supplementary Figure S3. Physical phenotyping of tofacitinib-treated and untreated *SOD1*^G93A^ mice stratified by sex.** Physical phenotypes were compared between untreated mice versus all six tofacitinib dose-regime combinations (**Fig 1A**) and by tofacitinib dose alone in male and female mice. **(A)** Body weight, **(B)** grip strength, **(C)** and rotarod phenotyping was performed every 2 weeks beginning at 60 days of age and analyzed via linear mixed model. Body weight is presented as percentage of bodyweight at 60 days of age and is normalized by cohort. Grip strength was normalized to weight. **(D)** Motor neurons were counted in stained lumbar spinal cord section and normalized by cohort. Neuromuscular junctions were counted in **(E)** gastrocnemius and **(F)** tibialis anterior sections. WT mice included for reference only and excluded from statistical analysis. *p < 0.05, **p < 0.01, ***p < 0.001, by linear mixed models (A-C) or Kruskal-Wallis (E-F). N = 129; 10-60 mice per group (A-C), N = 122; 5-58 mice per group (D-F).

**Supplementary Figure S4. Immune phenotyping in peripheral blood of tofacitinib-treated and untreated *SOD1*^G93A^ mice stratified by sex.** *SOD1*^G93A^ mice were sacrificed at end-stage disease and flow cytometry analyzed peripheral blood. Absolute immune counts were compared between untreated mice versus all six tofacitinib dose-regime combinations (**Fig 1A**) and by tofacitinib dose alone in male and female mice. **(A)** Complete blood count, **(B)** CD4+ T cells, **(C)** CD8+ T cells, **(D)** NK cells, **(E)** neutrophils, **(F)** Ly6c- monocytes, **G.** Ly6c+ monocytes. N = 63, 1-30 mice per group. WT mice included for reference only and excluded from statistical analysis. *p < 0.05, statistical significance by Kruskal-Wallis with post-hoc Dunn’s test with Bonferroni correction.

**Supplementary Figure S5. Neuroinflammation in the spinal cord of tofacitinib-treated and untreated *SOD1*^G93A^ mice stratified by sex.** *SOD1*^G93A^ mice were sacrificed at end-stage disease and flow cytometry analyzed spinal cord. Immune populations, assessed as a proportion of all CD45+ cells, were compared between untreated mice versus all six tofacitinib dose-regime combinations (**Fig 1A**) and by tofacitinib dose alone in male and female mice. **(A)** CD4+ T cells, **(B)** CD8+ T cells, **(C)** NK cells, (**D)** neutrophils, **(E)** monocytes, **(F)** microglia. **(G-I)** Activation marker surface expression was assessed on microglia by mean fluorescence intensity (MFI) as a fold-change over isotype-stained controls. **(G)** Forward scatter (FSC), **(H)** CD11c, **(I)** F4/80. WT mice included for reference only and excluded from statistical analysis. N = 63, 2-30 mice per group. Statistical significance by Kruskal-Wallis with post-hoc Dunn’s test with Bonferroni correction or by one-way ANOVA with post-hoc Tukey test as indicated by a Shapiro-Wilk test using p=0.05.

**Supplementary Figure S6. NK cell cytokine expression and STAT phosphorylation in response to multiple tofacitinib doses.** (**A**) NK-92 cells co-cultured with K-562 cancer cells and for four hours under one of four conditions: no tofacitinib, 15.62 ng/ml tofacitinib, 80 ng/ml tofacitinib (low-dose), or 530 ng/ml (high-dose). Cells were collected, RNA extracted, and cytokine gene expression was quantitated using qRT-PCR. Gene expression for IFN-γ (*ifng*), TNF-α (*tnf*), and IL-10 (*il10*) were assessed. (**B**) NK-92 cells were cultured overnight with IL-15 under one of four conditions: no tofacitinib, 15.62 ng/ml tofacitinib, 80 ng/ml tofacitinib (low-dose), or 530 ng/ml (high-dose). Cells were collected, lysed, and STAT3 and STAT5 phosphorylation were assessed via Western blot. n = 2-3 replicates for both assays.

**Figure S7. Principal component analysis of RNA-Seq by cohort, sex, and treatment regimen.** Plots before and after application of ComBat-Seq to correct for cohort effect. N = 58, 8-28 mice per group.

**Figure S8. Validation of RNA-Seq gene expression patterns.** RNA was extracted from fixed spinal cord sections of WT, untreated, and low-dose mice and analyzed using qRT-PCR. Expression of three of the top genes - *btnl10*, *ctsg*, *elane –* that were dysregulated in SOD1 but reversed by low-dose tofacitinib treatment were quantified. n = 2-3
